# Effects of the COVID-19 Pandemic on Primary Health Care for Chronic Conditions in Canada: Protocol for a Retrospective Pre-Post Study Using National Practice-Based Research Network Data

**DOI:** 10.2196/49131

**Published:** 2023-07-21

**Authors:** Michelle Howard, Kris Aubrey-Bassler, Neil Drummond, Marie-Thérèse Lussier, John A Queenan, Meredith Vanstone, Kathryn Nicholson, Amanda Ramdyal, Jennifer Lawson, Shuaib Hafid, Karla Freeman, Rebecca Clark, Dee Mangin

**Affiliations:** 1 Department of Family Medicine McMaster University Hamilton, ON Canada; 2 Primary Healthcare Research Unit Memorial University of Newfoundland St. John's, NL Canada; 3 Department of Family Medicine University of Alberta Edmonton, AB Canada; 4 Département de médecine de famille et de médecine d’urgence Université de Montréal Montréal, QC Canada; 5 Department of Family Medicine Queen's University Kingston, ON Canada; 6 Department of Epidemiology and Biostatistics Western University London, ON Canada

**Keywords:** COVID-19, chronic disease, primary health care, electronic health record, health services research, retrospective studies

## Abstract

**Background:**

Since the COVID-19 pandemic began, there have been concerns that interruptions to the health care system may have led to changes in primary care, especially for care of chronic conditions such as diabetes and heart failure. Such changes may have longer term implications for population health.

**Objective:**

This study aims to describe the impacts of the COVID-19 pandemic on indicators of primary care access, comprehensiveness, and appropriateness among adult patients, as well as on specific indictors of chronic conditions. Additionally, this study aims to determine whether any identified changes were associated with patient sociodemographic characteristics and multimorbidity.

**Methods:**

This is a retrospective, single-arm, pre-post study using Canadian Primary Care Sentinel Surveillance Network (CPCSSN) data. CPCSSN is a research network supported by a primary care electronic medical record database, comprising over 1500 physicians and nearly 2 million patients. We are examining changes in care (eg, frequency of contacts, laboratory tests and investigations, referrals, medications prescribed, etc) among adults. We will also examine indicators specific to evidence-based recommendations for care in patients with diabetes and those with heart failure. We will compare rates of outcomes during key periods of the pandemic between March 13, 2020, and December 31, 2022, with equal time periods before the pandemic. Differences will be examined among specific subgroups of adults, including by decade of age, number of comorbidities, and socioeconomic status. Regression models appropriate to outcome distributions will be used to estimate changes, adjusting for potential confounders. This analysis is part of a mixed-methods study with a qualitative component investigating how patients with diabetes with or without concurrent heart failure perceived the impact of the pandemic on access to primary care and health care–related decisions. This study was approved by the Hamilton Integrated Research Ethics Board (14782-C).

**Results:**

The start date of this study was October 5, 2022, and the prospective end date is January 31, 2024. As of May 2023, the study cohort (n=875,934) is defined, data cleaning is complete, and exploratory analyses have begun. Extended analyses using 2022 data are planned once the new data becomes available. We will disseminate results through peer-reviewed publications and academic conference, as well as creating evidence briefs, infographics, and a video for policy maker and patient audiences.

**Conclusions:**

This study will investigate whether the COVID-19 pandemic has resulted in changes in the provision of primary care in Canada and whether these potential changes have led to gaps in care. This study will also identify patient-level characteristics associated with changes in care patterns across the COVID-19 pandemic. Indicators specific to chronic conditions, namely diabetes and heart failure, will also be explored to determine whether there were changes in care of these conditions.

**Trial Registration:**

ClinicalTrials.gov NCT05813652; https://clinicaltrials.gov/ct2/show/NCT05813652

**International Registered Report Identifier (IRRID):**

RR1-10.2196/49131

## Introduction

The presence of strong primary care systems has been shown to be associated with superior and more equitable health outcomes in the population [1-6]. Core elements of primary care that contribute to population health outcomes include access, continuity, comprehensiveness, coordination, person focus, and equity enhancement [7]. Chronic diseases are common and represent the leading causes of death worldwide [8]. The core elements of primary care make this setting ideal for managing chronic diseases [4,9], and family medicine and other primary care services provide the majority of care for chronic diseases across the lifespan [4,10]. With increasing age, there is an increasing prevalence of multiple chronic diseases [11], and these patients require careful management to reduce morbidity and mortality and improve quality of life [12]. Life-limiting chronic diseases such as chronic obstructive pulmonary disease, heart failure, and chronic kidney disease [13-15] affect millions of people in Canada and are mostly managed in primary care [16-19]. Other conditions that are risk factors for numerous cardiovascular and cerebrovascular diseases (eg, diabetes and hypertension) are associated with poor health outcomes, and these are also managed in primary care [20].

On March 11, 2020, the World Health Organization declared the circulating SARS-CoV-2 as a pandemic. The SARS-CoV-2 pandemic placed a substantial strain on health care services for care of both COVID-19 illness (the majority of which was provided in primary care without need for hospitalization) and non–COVID-19 illness [8,21]. The documented early impacts of the COVID-19 pandemic were reductions in the number of patients visiting primary care offices [22], dramatic increases in remote care especially for older patients [22,23], and new strategies to maintain care such as implementing wellness checks for high-risk patients [24,25].

Early in the pandemic, there were reductions in visits for chronic condition management [20,26,27], preventive screening and diagnostic testing [28,29], and changes in physician referral behaviors [30]. The limited availability of testing and coordination of prevention services was a concern among Canadian family physicians [31]. Professional organizations recommended continuation of in-person care during the pandemic for complex chronic conditions and when physical examination was required; however, many patients avoided seeking primary care, and clinics experienced decreased attendance relating to the care of serious conditions [32,33]. Although telemedicine strategies can be effective for some chronic disease monitoring, patients who experience lack of equity in access to health care are also likely to experience barriers to using technology [34-36].

The onset of the pandemic worsened existing challenges to the prospects of achieving and maintaining an adequate level of comprehensive primary care in Canada. Understanding the ways in which the core functions of primary care [37-39] were affected by the COVID-19 pandemic is essential for chronic condition management and for system assessment overall. Although there are reports that the volume of primary care in Canada has largely returned to prepandemic levels, it is not clear whether and for whom there is a need to “catch up” on care for chronic diseases. For practices with a tendency for overuse of testing and investigations, these may have declined to more appropriate levels during the pandemic, also highlighting the areas of practice improvement. The objectives of this study are to describe the impacts of COVID-19 on chronic disease care and to determine whether changes were associated with patients’ sociodemographic characteristics and multimorbidity.

## Methods

### Data Source

The Canadian Primary Care Sentinel Surveillance Network (CPCSSN [40]) is a multidisease electronic medical record (EMR) research and surveillance data repository that securely collects deidentified health information from EMRs of participating primary care clinicians (eg, family physicians and nurse practitioners) who are referred to as “sentinels.” CPCSSN currently brings together 13 primary care research networks for the purposes of research, disease surveillance, and quality improvement, with more networks due to join in the near future. Every 6 months, CPCSSN acquires deidentified patient-level clinical data from primary care EMR systems held by 1583 primary care providers across Canada, with approximately 200 patient encounters per month per physician [20]. CPCSSN acquires most of the data fields in Canadian primary care EMRs with the exceptions of portable document format such as imaging data and the narrative “Subjective, Objective, Assessment, and Planning” notes. The acquired data are processed into a standard format and are made available for surveillance and research [41]. The relative frequency, longitudinal format, and comprehensiveness of CPCSSN data are ideal for the study of changes in practice, through time, in response to significant environmental imperatives, such as public health or research initiatives [41]. Numerous case definition validation studies have been completed [42-44], and the data have also been used to evaluate patterns and quality of health care [19,45].

### Study Design

This protocol was designed using the RECORD (REporting of studies Conducted using Observational Routinely collected Data) statement [46]. We will conduct a retrospective closed cohort study with a single group using a pre-post design. We will examine prevention and management activities for exemplar conditions on the basis of having a validated CPCSSN case definition [47,48] (ie, diabetes and heart failure), having substantial prevalence in primary care, and either having high morbidity or being risk factors for morbidity if not well managed. Patient-level data extracted from the CPCSSN repository between 2018 and 2022 will be used to investigate changes in rates of presentation and clinical management of various chronic diseases before, and through the phases of, the pandemic.

### Study Population

The study population is all patients who were 18 years and older in 2018, and some analyses will focus on subgroups of patients with specific chronic conditions. We will use the 2-year contact population as recommended previously for defining practice populations for primary care [49]. This method uses contacts within encounter, medication, and billing-related data tables, and has been used previously with CPCSSN data to produce national chronic disease estimates [50]. This method will be used in the period from March 13, 2018, to March 13 2020. We will use this population rather than the full CPCSSN patient population data (dating back more than 2 decades for some networks) to limit the size of the data set, while maintaining the patient population that is likely to have a relationship with a primary care practice during the study period. Although this approach may exclude patients who are younger, healthier, and less likely to seek primary care compared with patients with chronic conditions, we do not expect this to have a major impact on findings because this study focuses on the age group where chronic condition prevalence begins to increase and where screening guidelines apply (eg, diabetes). Therefore, this age group is more likely to see a primary care physician regularly.

People who died during the study period will be included up to the point of death using the death date if captured or the presence of a deceased status with date estimated from the most recent contact in CPCSSN data. Patients who were older than 105 years at the start of the COVID-19 pandemic will be excluded as a possible data error.

### Variables of Interest

#### Primary Exposure

The primary exposure is the start of the pandemic in March 13, 2020. This date was selected as it marks the beginning of Canadian provincial governments responding to the formal declaration of the pandemic by the World Health Organization on March 11, 2020, by enforcing sweeping public health and safety measures. The prepandemic time frame used is defined as June 22, 2018, through March 12, 2020. The pandemic time frame used in this study runs from March 13, 2020, through December 31, 2022. In addition to comparison between these 2 periods, 30-day interval rates will also be calculated for selected outcomes and indicators where possible.

#### Outcome Definitions and Measures

Outcomes were informed by a large primary care trial on improving prevention and management of chronic conditions in primary care [51], a quality index created for primary care with items measurable in EMRs [52], and consideration of data capture across the networks of CPCSSN. We further conceptualized outcomes according to whether they represent aspects of access, comprehensiveness, or appropriateness (Table 1).

**Table 1:** Outcomes and outcome measures.

Population		Indicator		Related domain		Data source^a^
**Number and types of encounters**						Encounter, encounter diagnosis, and billing
	All patients in our defined cohort	Overall number of all encounters	Access			
	Patients with the CPCSSN^b^ case definition of diabetes and HF^c^	Overall number and types of diabetes-related and HF-related investigations	Comprehensiveness			
**Diagnoses addressed in encounters**				Comprehensiveness and appropriateness		Encounter, encounter diagnosis, and billing
	All patients	Number of different diagnoses addressed per patient per year				
	Patients with ≥1 condition with the validated CPCSSN case definition	Number and proportion of chronic conditions (ie, CPCSSN case definitions) addressed by physician at least once a year				
	Patients with the CPCSSN case definition of diabetes and HF	Overall number and types of diabetes-related and HF-related investigations				
	Patients with the CPCSSN case definition of diabetes and HF	Proportion of patients with diabetes-related encounter and HF-related encounter every 6 and 12 months (as appropriate for condition)				
**Services provided**				Comprehensiveness		Billing, vaccine, and medication
	Patients age ≥65 years	Number of patients receiving pneumococcal vaccine and influenza vaccine				
	Patients with the CPCSSN case definition of diabetes and HF	Proportion of patients with influenza vaccination				
**Procedures performed**				Appropriateness		Billing
	Patients with the CPCSSN case definition of diabetes	Proportion of patients with foot examinations in last 12 months				
**Physical examinations**				Appropriateness		Examinations
	All patients in our defined cohort; all patients grouped by CPCSSN case definition	Proportion with BP^d^ measured every 6 months or every 12 months (as appropriate for condition)				
**Referrals made to specialists**				Comprehensiveness		Referral
	All patients in our defined cohort; all patients grouped by CPCSSN case definition	Overall number and types of referrals; proportion referred to cardiology, endocrinology (as appropriate for condition)				
**Investigations performed**				Appropriateness		Laboratory
	All patients in our defined cohort	Number of vitamin D, CBC^e^, eGFR^f^, ACR^g^, and liver function tests performed				
	Patients with CKD^h^	Number with and frequency of eGFR and ACR tests				
	Patients without diabetes	Number with HbA_1c_^i^, FBS^j^ measured (screening)				
	Patients taking or not taking statins	Number with and frequency of lipid panel/LDL^k^				
	Patients taking or not taking levothyroxine	Number with and frequency of TSH^l^ measured				
	Patients with the CPCSSN case definition of diabetes	Proportion with HbA_1c_ measured every 6 months and every 12 months				
**Monitoring results**				Appropriateness		Examination
	All patients in our defined cohort; all patients grouped by CPCSSN case definition and age	Proportion with BP ≤140/90 or ≤130/80 (as appropriate based on chronic conditions and age); proportion of patients in the following sBP^m^ ranges: ≤130, 131-140, 141-150, 151-160, and >160				
**Medications**				Comprehensiveness and appropriateness		Medication and risk factor
	Patients with the CPCSSN case definition of diabetes	Proportion of patients with and overall number and frequency of metformin prescriptions, insulin prescriptions, and other oral antidiabetic prescriptions^n^				
	Patients with the CPCSSN case definition of HF	Proportion of patients with and overall number and frequency of β-blocker prescriptions, ARB^o^ prescriptions, and ACE-I^p^ prescriptions				
	Patients who smoke	Number of patients with NRT^q^ prescription				

^a^CPCSSN data table.

^b^CPCSSN: Canadian Primary Care Sentinel Surveillance Network.

^c^HF: heart failure.

^d^BP: blood pressure.

^e^CBC: complete blood count.

^f^eGFR: estimated glomerular filtration rate.

^g^ACR: albumin-to-creatinine ratio.

^h^CKD: chronic kidney disease.

^i^HbA_1c_: hemoglobin A_1c_.

^j^FBS: fasting blood sugar.

^k^LDL: low-density lipoprotein.

^l^TSH: thyroid-stimulating hormone.

^m^sBP: systolic blood pressure.

^n^Other oral antidiabetic medications include classes such as sodium-glucose cotransporter-2 inhibitors, sulfonylureas, meglitinides, dipeptidyl peptidase 4 inhibitors, etc.

^o^ARB: angiotensin receptor blocker.

^p^ACE-I: angiotensin-converting enzyme inhibitor.

^q^NRT: nicotine replacement therapy.

*Access* to the practice will be measured as the overall number of encounters.

*Comprehensiveness* refers to primary care addressing “all problems in the population (with short-term referral as needed), except those that are too unusual (generally a frequency of less than 1 or 2 per thousand in the population served) for the primary care practitioner or team to treat competently” [1]. As indicators of comprehensiveness, we will measure number of different diagnoses addressed per patient per year, proportion of patients’ chronic conditions addressed at least once a year, number of pneumococcal vaccines and influenza vaccines in patients 65 years or older, and number of prescriptions for nicotine replacement therapy

*Appropriateness* will be examined in 2 ways: laboratory tests that are indicated for monitoring of chronic conditions but may be ordered more frequently than necessary (eg, hemoglobin A_1c_ in diabetics) and laboratory tests that are frequently ordered routinely (or for screening purposes) but may be overused in certain populations, including thyroid function tests, vitamin D, albumin-to-creatinine ratio, and lipid panel testing [53]. We hypothesize that some of these tests, while indicated at a base frequency, may be commonly overused; thus, their frequency may have declined during the pandemic.

Diabetes and heart failure are 2 chronic conditions where aspects of care can be reasonably evaluated in CPCSSN. We will describe care across the population in terms of frequency of contacts, laboratory tests and investigations, and medications prescribed that would suggest care that is in step with evidence-based recommendations [54]. For patients with diabetes, we will measure the percentage of patients with a diabetes-related encounter every 6 and 12 months; percent with a foot examination in the last 12 months; percent with a blood pressure measure and hemoglobin A_1c_ test every 6 and 12 months (and results); percent with influenza vaccination; overall number of diabetes-related investigations; percent referred to endocrinology; percent receiving, and number and frequency of metformin, insulin, and other antidiabetic prescriptions. For patients with heart failure, we will measure percentage of patients with heart failure–related encounters every 6 and 12 months; number of investigations (echocardiogram and blood tests); percent with a blood pressure measure every 6 and 12 months (and results); percent with influenza vaccination; percent receiving β-blocker, angiotensin-converting enzyme inhibitors, and angiotensin receptor blockers; and percent referred to cardiology and other specialists.

### Statistical Analysis and Power

As of 2018, there were 1.13 million adult patients in the CPCSSN database, with 65% (734,500/1,130,000) having at least 1 chronic condition [55]. A sample size of 1276 paired (pre-post) individuals would be required to detect a change from 50% to 55% of patients experiencing an outcome, with a correlation between paired observations of 0.2 [56]. With a correlation between pairs of 0.1, the required sample size would be 1432. We expect to have adequate sample size to examine changes within specific conditions given the approximate prevalence of hypertension (22%), diabetes (10%), chronic obstructive pulmonary disease (4%), and heart failure (4%) among adults in CPCSSN data or other Canadian data [13,55].

#### Descriptive Analyses

To describe the cohort, continuous or count variables will be analyzed for their mean, median, SDs, and IQRs. For categorical variables, percentages will be calculated. We will present outcomes stratified by sex, age category (40-49, 50-59, 60-69, and ≥70 years), urban versus rural patient address, and neighborhood income quintile (1-2 [lower] vs 3-5 [higher]).

#### Analyses of Changes Over Time

We will present the proportions of patients experiencing the outcomes for each time period or mean changes for continuous variables and 95% CIs. Statistical significance of changes from pre to post time period will be assessed using the McNemar test for binary variables, the Wilcoxon signed rank test for original variables, and the paired *t* test for continuous variables. Regression models appropriate to outcome distributions will be used to control for potential confounders of age (<70 vs ≥70 years), sex, count of comorbidities (CPCSSN-validated conditions), neighborhood income quintile, and CPCSSN network (corresponding to location within Canada). Interactions will be examined in the descriptive analysis and will be considered for inclusion in models if they are found to be relevant. We will present adjusted estimates and 95% CIs for outcomes and parameter estimates for the independent variable and covariates. Analyses will be completed using SAS (version 9.4; SAS Institute, Inc).

#### Sensitivity Analysis

To examine whether results are biased because of the nonrepresentativeness of the CPCSSN population, we will conduct the analyses on a sample of patients that is representative of the Canadian population based on age, sex, neighborhood socioeconomic status, and rural or urban residence.

### Ethics Approval

This study was approved by the Hamilton Integrated Research Ethics Board on April 11, 2022 (project number 14782-C). Because we will be using deidentified data, no consent is required. This study is also registered with ClinicalTrials.gov (NCT05813652).

## Results

### Study Timeline

The start date of this study was October 5, 2022, and the prospective end date is January 31, 2024. At the time of writing (May 2023), we have finished data cleaning and begun exploratory analyses. As there are known consistencies with CPCSSN data, having a longer exploratory analysis phase has allowed us to liaise with central CPCSSN data managers in order to prepare data for the main analyses. We have now also defined our study cohort: a total of 875,934 individuals between the ages of 18 and 105 years as of 2018 were included.

### Future Analyses

We will attempt to extend our analyses to include the first half of 2022 when data become available if our timelines and resources allow. At the time of writing this protocol (May 2023), data were available up to December 2021. The additional 2022 data will allow us to investigate changes in primary care in the later portion of the COVID-19 pandemic, capturing the Omicron wave, and measure whether any changes identified in the earlier portion of the pandemic were sustained or returned to prepandemic patterns of care.

### Dissemination

We plan to share knowledge products for patients and the public with the patient advisory committee being created as part of the David Braley Primary Care Research Collaborative at McMaster University, with whom several authors are affiliated. *For clinicians*, we will create evidence briefs to be shared with the national and provincial colleges of family physicians through social media and our CPCSSN practice-based research network linkages. *For researchers and policy and practice decision makers* (eg, College of Family Physicians of Canada), we will produce peer-reviewed publications and evidence briefs where we will contextualize the new knowledge against recent research relating to pandemic recovery in health care. The 1- to 2-page evidence briefs will be shared with knowledge users involved in the development and implementation of policies and resources related to pandemic recovery. *For patients*, we will create infographics and a short video that will raise awareness of the issue of chronic condition care gaps and describe patients’ perspectives. These will be circulated via social media and clinical organizations.

## Discussion

### Key Findings

The aim of this study is to help identify signals of change across several domains of primary care, including access, comprehensiveness, and appropriateness, that may have occurred in response to the COVID-19 pandemic. This will be done by investigating the overall number of primary care encounters during the pandemic compared with prepandemic, as well as potential changes in the number of diagnoses addressed during encounters, number of blood pressure measurements, number of vaccines provided, number of blood tests carried out, and so on. Potential changes in indicators specific to the management of chronic conditions will also be investigated, for example, frequency of HbA_1c_ testing for those with diabetes, number and frequency of visits for monitoring of heart failure, and number of prescriptions for diabetes or heart failure. Potential changes in any of these indicators may indicate that gaps in care are present that may benefit from targeted recovery measures.

However, these potential changes in primary care are not inherently negative. For example, many blood tests, examinations, and procedures are considered overused in certain populations and may be conducted more frequently than necessary [53,57,58]. If decreases in number or frequency of these overused tests are identified during the pandemic, this may be considered more appropriate use when compared to prepandemic levels. Furthermore, decreases in frequency of certain primary care interventions may not be clinically significant or necessarily lead to negative health outcomes. Therefore, it is important that each identified change be carefully considered in a broad clinical context before firm conclusions can be drawn.

### Strengths and Limitations

The strengths of this study include the use of a large, multiprovince primary care EMR data source, developed to understand the epidemiology of chronic diseases and care in Canadian primary care. The data set is regularly updated and includes coded information on most aspects of primary care practice. The relative currency, longitudinal format, and comprehensiveness of CPCSSN data are ideal for the study of practice changes, through time, in response to significant environmental imperatives, such as the sweeping public health measures put in place during the pandemic [41]. Numerous case definition validation studies have been completed [42,43], and the data have also been used to evaluate patterns and quality of health care [19,45]. CPCSSN has undertaken extensive data cleaning algorithms to address data quality issues [41].

There are limitations in this study. EMR data are collected for clinical and administrative purposes and are not primarily collected for research, which may result in incomplete capture of information. Furthermore, participating sentinel clinics are overrepresented by academic practices [59], and by extension, we cannot be certain to what extent the patients in the CPCSSN data differ from other Canadian primary health care patients. There may be issues of nonparticipation bias and variation in data quality. This study will not provide information on people who are not attached to a primary care provider. As with primary care patients generally, older adults and female individuals are overrepresented in the CPCSSN database as compared with the national population; however, as this study is about changes in care patterns, this data set is representative of the population of interest [59]. Clinical context for some research based on secondary use of EMR data can be difficult to infer. Detailed information on family and living circumstances, ethnicity, language spoken, education, and socioeconomic status is rarely captured in EMRs, so it is more difficult to conduct equity analyses, relying instead on neighbor-level indicators.

### Patient and Public Involvement

This research was initially designed and received funding without patient involvement. When the research team presented the data request to the CPCSSN data access committee (May 2022), a patient advisor on the committee provided input. The advisor noted that the proposal was broad in terms of the scope of chronic diseases to be investigated. In response, we refined our analysis plans to examine diabetes and heart failure in greater detail as exemplar diseases. The advisor also suggested that it would be important to consider the changes in the health care system and behaviors of the public over different phases of the pandemic, especially since the Omicron variant.

### Conclusions

We anticipate that this study will provide evidence that will inform whether and in what aspects of primary care the COVID-19 pandemic is associated with interruptions that may require attention. It will also highlight gaps or provide reassurance with respect to chronic condition care for populations who were expected to be disproportionately affected by pandemic health care changes such as those who are older or of lower socioeconomic status.

## Appendix

Multimedia Appendix 1 Peer review report by Canadian Institutes of Health Research (Canada).

## Abbreviations

CPCSSN: Canadian Primary Care Sentinel Surveillance Network
EMR: electronic medical record
RECORD: REporting of studies Conducted using Observational Routinely collected Data

## Data Availability

The data set from this study is held securely by CPCSSN, and data sharing agreements prohibit the authors from making the data set publicly available. Researchers can request access to CPCSSN data on their website [60].
