# Supplementary material for: Effects of the COVID-19 Pandemic on Primary Health Care for Chronic Conditions in Canada: Protocol for a Retrospective Pre-Post Study Using National Practice-Based Research Network Data
Source: JMIR Res Protoc. 2023 Jul 21;12:e49131. doi: 10.2196/49131 (PMC10403797; doi:10.2196/49131)
Supplement: Multimedia Appendix 1 [file resprot_v12i1e49131_app1.pdf]

February/Février 28, 2022

DC0190GP

# Canadian Institutes of Health Research / Instituts de recherche en santé du Canada

## Notice of Decision / Avis de décision

Application Number/Numéro de la demande: 478202
Committee Code/Code du comité: WHI

Applicants/Candidats: Dr. Michelle Isabel Howard, Dr. Derelie Mangin

With/Avec: Dr. F. Aubrey-Bassler, Dr. N. Drummond, Dr. S. Hosseini, Dr. K. Nicholson, Dr. J. Queenan, Dr. A. Ramdyal, Dr. M. Vanstone

Institution paid/ McMaster University

Title/Titre: Impacts of the COVID-19 pandemic on the primary care of chronic conditions

Primary Inst./ Inst. principal: Health Services and Policy Research / Services et politiques de la santé

Other Related Inst./ Autres inst. connexes:

**Competition Outcome/Résultats du concours:** O. Gr.: Addr. the Health Impacts of COVID-19 - Imp. of def/disp care on health / Subv. de Fon: Étude réper. glob. de COVID sur santé - Conséq report/dép de santé

November/Novembre 08, 2021

**Number in competition/Nbre de demandes dans le concours:** 58

**Number approved/Nbre de demandes approuvées:** 41

**Decision on your application/ Décision sur votre demande:** Approved / Approuvée

**Average annual amount/ Montant annuel moyen:** $142,285

**Term/Durée:** 2 **yrs/ans** 0 **months/mois**

**Peer Review Committee Recommendation, for your information and use/ Recommandation du comité d'examen par les pairs, pour fins d'information et d'utilisation:**

**Committee/Comité:** Operating Grant : Addressing the Wider Health Impacts of COVID- / Sub. de fonct.:Étude des répercussions globales de la COVID 19 sur la santé

**Application rank within the competition/** 35

**Percent Rank Within the Competition/** 60.34%

**Rating/** 3.73

**Recommended average annual amount/ Montant annuel moyen recommandé:** $142,285

*** Applications receiving a score of less than 3.5 on any evaluation criteria will not be considered for Funding. / Les demandes qui ont reçu une note inférieure à 3.5 pour n'importe quel des critères d'évaluation ne sont pas admissibles.

This document is for information only. Official payment is stated on the CIHR Authorization for Funding.
Document à titre d'information seulement. Le paiement officiel est indiqué sur l'autorisation de financement des IRSC.

Canadian Institutes of Health Research
160 Elgin Street, 9th Floor
Address Locator 4809A
Ottawa, Ontario K1A 0W9

Instituts de recherche en santé du Canada
160, rue Elgin, 9e étage
Indice de l'adresse 4809A
Ottawa (Ontario) K1A 0W9

Institute of Aging
Institute of Cancer Research
Institute of Circulatory and Respiratory Health
Institute of Gender and Health
Institute of Genetics
Institute of Health Services and Policy Research
Institute of Human Development and Child and Youth Health
Institute of Indigenous Peoples' Health
Institute of Infection and Immunity
Institute of Musculoskeletal Health and Arthritis
Institute of Neurosciences, Mental Health and Addiction
Institute of Nutrition, Metabolism and Diabetes
Institute of Population and Public Health

Institut du vieillissement
Institut du cancer
Institut de la santé circulatoire et respiratoire
Institut de la santé des femmes et des hommes
Institut de génétique
Institut des services et des politiques de la santé
Institut du développement et de la santé des enfants et des adolescents
Institut de la santé des Autochtones
Institut des maladies infectieuses et immunitaires
Institut de l'appareil locomoteur et de l'arthrite
Institut des neurosciences, de la santé mentale et des toxicomanies
Institut de la nutrition, du métabolisme et du diabète
Institut de la santé publique et des populations

February 28, 2022

Dr. Michelle Isabel Howard
McMaster Health Campus
Department of Family Medicine, McMaster University
100 Main Street West, 5th Floor
Hamilton, Ontario L8P 1H6

Dear Dr. Howard,

On behalf of the Government of Canada, the Canadian Institutes of Health Research (CIHR) is pleased to inform you that your recent application "Impacts of the COVID-19 pandemic on the primary care of chronic conditions", submitted to the Operating Grant: Addressing the Wider Health Impacts of COVID-19, has been approved for funding.

As a successful nominated principal applicant (NPA), please note that there are additional Conditions of Funding outside of CIHR's standard requirements related to this competition (please refer to the Conditions of Funding section of the funding opportunity and your Authorization for Funding form (AFF) for more details). Furthermore, if you are in receipt or become eligible to receive any funding from another source for any part of this project, you must advise CIHR immediately by following the instructions outlined in the "Funding Overlap Declaration" form (https://cihr-irsc.gc.ca/e/797.html). Failure to self-declare overlap could lead to CIHR cancelling all funding related to this grant.

The decisions of this funding opportunity are under embargo. CIHR Communications will be in touch with you in the near future regarding plans for an official public announcement of the decisions. We would ask grant recipients and their institutions to hold off on any public communications (e.g., news releases, external web postings, social media) until the official announcement has taken place.

As outlined in CIHR's equity strategy (https://cihr-irsc.gc.ca/e/50068.html), CIHR is committed to creating an equitable funding system by identifying and eliminating systematic biases towards any individual or group that would hinder access to CIHR funds. CIHR committed to ensuring a minimum proportion of funding was allocated to applications related to Indigenous Health Research (IHR) and that the proportion of grants awarded to applications submitted in French and applications submitted by female nominated principal applicants was not less than the proportion of applications submitted by those groups. However, for this competition, the proportion of applications for all groups was met without the need to apply the equalization process.

As CIHR does not notify co-applicants of the decision, we ask that you inform those individuals involved, along with their research institutions (if different from your own), of the outcome of this application.

Please note that NPIs may be contacted by CIHR to complete a short survey regarding barriers and enablers to their research. CIHR is committed to promoting opportunities for knowledge sharing and collaboration to facilitate and accelerate Canada's research response to the COVID-19 outbreak. As such, the results of the survey may be used to support future activities related to knowledge mobilization in the COVID-19 context.

CIHR would like to remind grant recipients that all COVID-19 related publications must be open access, in alignment with the call from the Chief Science Advisors (https://www.ic.gc.ca/eic/site/063.nsf/eng/h_98016.html), and that all data produced as a result of this funding must be shared in line with the Joint statement on sharing research data and findings relevant to the novel coronavirus (nCoV) outbreak (https://wellcome.org/coronavirus-covid-19/open-data). Data related to research by and with First Nations, Métis, or Inuit communities whose traditional and ancestral territories are in Canada must be managed in accordance with data management principles developed and approved by those



CIHR IRSC
Discoveries for life / Découvertes pour la vie
Canada

communities, and on the basis of free, prior and informed consent. This includes, but is not limited to, considerations of Indigenous data sovereignty, as well as data collection, ownership, protection, use, and sharing.

Should you require additional information, please contact CIHR at EHTRF-FRNMS@cihr-irsc.gc.ca.

Sincerely,

Tammy Clifford, PhD
Vice-President, Research Programs
Research Programs Portfolio

**Review Type/Type d'évaluation:** Committee Member 1/Membre de comité 1
**Name of Applicant/Nom du chercheur:** Howard, Michelle Isabel
**Application No./Numéro de demande:** 473638
**Agency/Agence:** CIHR/IRSC
**Competition/Concours:** 2021-11-08 Operating Grant: Addressing the Wider Health Impacts of COVID-19/Subvention de fonctionnement : Étude des répercussions globales de la COVID 19 sur la santé
**Committee/Comité:** Operating Grant : Addressing the Wider Health Impacts of COVID-/Sub. de fonct.:Étude des répercussions globales de la COVID 19 sur la santé
**Title/Titre:** Impacts of the COVID-19 pandemic on the primary care of chronic conditions

---

**Assessment/Évaluation:**

The project focuses on the impact of the pandemic on primary care access for patients with chronic diseases. The project is a mixed method design, appropriate to the research questions. The team is well aligned to address the research aims and methodological approach. The team recognizes that the data set does not allow for a robust analysis of demographic data related to marginalized populations and only make inferences with the qualitative data. The team has the expertise to carry out the project and letter of reference provided by the Canadian Primary Care Sentinel Survellaince Network was critical to the success of the quantitative component. Overall the project aligns with the funding opportunity but there should be more specific attention to marginalized populations. The budget is very reasonable given the work the team wants to undertake.

**Review Type/Type d'évaluation:** Committee Member 2/Membre de comité 2
**Name of Applicant/Nom du chercheur:** Howard, Michelle Isabel
**Application No./Numéro de demande:** 473638
**Agency/Agence:** CIHR/IRSC
**Competition/Concours:** 2021-11-08 Operating Grant: Addressing the Wider Health Impacts of COVID-19/Subvention de fonctionnement : Étude des répercussions globales de la COVID 19 sur la santé
**Committee/Comité:** Operating Grant : Addressing the Wider Health Impacts of COVID- /Sub. de fonct.:Étude des répercussions globales de la COVID 19 sur la santé
**Title/Titre:** Impacts of the COVID-19 pandemic on the primary care of chronic conditions

---

**Assessment/Évaluation:**

Summary of the application:

People with chronic conditions cannot access their family doctors for routine check-ups due to the COVID-19 pandemic. In avoiding the spread of the virus, most primary care has shifted to virtual care and in-person visits have reduced, which might have a negative impact on the management of chronic conditions. Given the situation, this study aims to evaluate the impacts of COVID-19 on chronic disease care using a large database with electronic medical record data from over 1500 family doctors' practices across Canada. This study will provide evidence on which specific aspects of primary care have been most impacted in the pandemic, and it will identify possible solutions to be recommended for recovering from the pandemic.

Strengths:

- Clear justification for the relevance and importance of the project.
- Research questions and objectives of the proposed study are clear.
- A clear definition of Therapeutic Recreation (TR) was provided.
- Literature review is relevant.
- Potential of the proposed research to achieve timely impacts and maximize health benefits within a rapid response timeframe.
- Data source CPCSSN is well introduced
- Sampling and recruitment, data collection, analysis and integration are well-described.
- The team has strong expertise in different areas that are relevant to the study.

Weakness:

- Additional literature on the impact of the COVID-19 on primary care and the negative impact on the accessibility of health care for different population, and clearly indicate the knowledge and service gaps would be beneficial for the proposed study.
- I would suggest providing a clear definition of conditions management as the research background.
- Whether the different types of data collection have influences on the quality of data and how to address the challenges.
- Gender could be strengthened in this application – in background, recruitment, data analysis, and KT.

**Review Type/Type d'évaluation:** Committee Member 3/Membre de comité 3
**Name of Applicant/Nom du chercheur:** Howard, Michelle Isabel
**Application No./Numéro de demande:** 473638
**Agency/Agence:** CIHR/IRSC
**Competition/Concours:** 2021-11-08 Operating Grant: Addressing the Wider Health Impacts of COVID-19/Subvention de fonctionnement : Étude des répercussions globales de la COVID 19 sur la santé
**Committee/Comité:** Operating Grant : Addressing the Wider Health Impacts of COVID-/Sub. de fonct.:Étude des répercussions globales de la COVID 19 sur la santé
**Title/Titre:** Impacts of the COVID-19 pandemic on the primary care of chronic conditions

---

**Assessment/Évaluation:**

**Proposed research**: In the proposed research, the applicants will conduct a database analysis and interviews to understand the impacts of COVID-19 on chronic disease care.

**Impact of research**: This work seems very important given the duration of the impacts of the COVID-19 pandemic and to date there hasn't been work in this area.

Strengths
- The proposed methods: a concurrent mixed-method explanatory design are suitable and well described.
- The authors will use CPCSSN for their database analysis and to recruit the qualitative sample through the participating primary care physicians.
- Both sex and gender analysis are planned. The analysis are well described and thoughtfully presented.
- KT involves outputs for various stakeholders including patients, clinicians, and decision makers.
- Data integration process is clear and well described.

Weaknesses
- The notion of 'catching up' on primary care of conditions interrupted by the pandemic is mentioned in the background and is really interesting but isn't well explored in the rest of the proposal. It would have been interesting to link this to the KT question.
- The KT research question could have been more specific, as it currently states: *What recommendations for primary care can be made to inform and prioritize strategies for sustained chronic condition management?* – this does not link at all to the pandemic.
- It wasn't totally clear whether interrupted time series analysis would take place as they applicants state they will explore the merit of such analysis.
- It would be helpful for the authors to describe information power and how they will know when they have reached it.

Applicants: The NPA and co-PA have an established track record of research grants and outputs. The team is from Ontario, Newfoundland, and Alberta with early, mid-career and established researchers.

Budget: Budget is reasonable although there are no trainees included on the grant.
